# Translational stewardship in pandemic therapeutics: ivermectin, evidentiary misalignment, and the propagation of constraints

**DOI:** 10.1186/s12967-026-08595-z

**Published:** 2026-07-09

**Authors:** Harlan Breindel

**Affiliations:** Massachusetts Institute of Technology, Cambridge, USA

**Keywords:** Translational medicine, Translational stewardship, Evidentiary misalignment, Drug repurposing, Pharmacokinetics, Ivermectin, COVID-19, Evidence synthesis, Infodemic, Knowledge translation

## Abstract

The COVID-19 pandemic exposed a recurring vulnerability within translational medicine: early therapeutic signals can acquire clinical, public, and institutional authority before the evidentiary pathway has established pharmacologic plausibility, evidence quality, clinical efficacy, and appropriate use. This Commentary proposes **translational stewardship** as an upstream response. Its central claim is that positive therapeutic signals and their critical plausibility constraints should co-travel through the translational pathway. A positive biological signal and a clinically plausible therapeutic signal are different evidentiary objects; when that distinction is lost, perceived therapeutic readiness can exceed evidentiary maturity. Ivermectin provides a concentrated case study. An initial in vitro antiviral finding generated a legitimate but low-confidence repurposing signal, while subsequent pharmacokinetic and pharmacodynamic analyses substantially constrained the plausibility of direct antiviral efficacy at clinically achievable exposures. Early clinical evidence was heterogeneous and fragile, portions of the evidence base were later affected by data-integrity concerns, and larger randomized trials did not demonstrate clinically meaningful benefit. Nevertheless, ivermectin acquired credibility through unstable evidence synthesis, clinical advocacy, public amplification, and institutional-distrust narratives. The broader lesson is not that early signals should be suppressed, but that critical constraints should be encoded, preserved, and propagated before provisional findings acquire durable therapeutic authority.

## Abstract (significance)

During the COVID-19 pandemic, some early treatment signals gained public and clinical attention before the evidence was strong enough to support practical use. This article uses ivermectin as a case study to show how early laboratory findings can become separated from the pharmacologic and clinical constraints needed to interpret them. It argues that translational medicine needs stronger stewardship practices so that limitations, uncertainty, and plausibility constraints travel alongside promising signals before those signals acquire durable therapeutic authority.

## Introduction

The COVID-19 pandemic made visible a translational problem that extends beyond any single therapeutic candidate. Under conditions of urgency, early-stage therapeutic signals can acquire practical authority before the evidentiary pathway has established whether they are pharmacologically plausible, clinically effective, supported by adequate evidence, and appropriate for use. Drug repurposing was a necessary part of the pandemic response; existing compounds with known safety profiles or plausible mechanisms deserved rapid assessment. The vulnerability arose when preliminary findings traveled faster than the constraints needed to interpret them.

That asymmetry matters because downstream correction is difficult once a therapeutic claim has acquired visibility, constituencies, and explanatory narratives. Much post-pandemic work rightly focuses on countering misinformation and improving public understanding of science after misleading claims enter public discourse [1, 2]. Such work remains important. The problem for translational medicine is that some claims acquire authority before correction can operate. Advocacy networks, unstable evidence syntheses, clinical protocols, media platforms, and institutional-distrust narratives can together create parallel systems of validation in which later evidence struggles to restore proportion.

This Commentary therefore looks upstream, to the point at which therapeutic signals first acquire credibility and investigative momentum. Its central claim is that translational stewardship should make positive signals and their critical plausibility constraints co-travel through the evidentiary pathway. A positive biological signal is not the same evidentiary object as a clinically plausible therapeutic signal. By the same logic, an exposure-feasibility analysis that materially lowers the likelihood of clinical translation is not merely a caveat; it is a translational finding. Stewardship means encoding those constraints before a preliminary signal circulates as therapeutic promise.

Four linked concepts organize the argument. Translational vulnerability describes the susceptibility of early-stage findings to premature operationalization. Constraint separation occurs when positive signals become detached from the limiting conditions needed to interpret them. That separation can enable parallel validation: the accumulation of practical authority outside the ordinary evidentiary pathway. The result is translational misalignment, in which perceived therapeutic readiness exceeds evidentiary maturity. Translational stewardship names the proposed response: encoding, preserving, and propagating critical plausibility constraints alongside therapeutic signals.

In translational-stage terms, misalignment occurs when a claim is used as though it occupies a later position in the pathway than the evidence supports. A T1 biological signal may be treated as T2 clinical support; unstable T2 evidence may become a T3 practice recommendation; or public-health behavior may emerge before guideline, regulatory, or population-level assessment has occurred [3]. Ordinarily, time, peer review, clinical evaluation, evidence synthesis, and guideline appraisal help maintain these distinctions. Under pandemic conditions, those buffers weakened.

The article uses ivermectin as a concentrated case rather than as an end in itself. Its value lies in convergence. A legitimate in vitro signal, major pharmacologic constraints, fragile early clinical evidence, unstable synthesis practices, public demand, and large-scale amplification all operated in the same episode. Pandemic conditions compressed discovery, evaluation, communication, and uptake, allowing credibility to accumulate faster than evidentiary maturity.

This framing draws on infodemic scholarship, the Social Amplification of Risk Framework, and translational-stage models. Infodemic work explains how rapidly circulating information environments complicate public-health decision-making [1, 2]. Risk-amplification scholarship shows how social, institutional, and media processes can alter perceptions beyond technical assessment alone [4]. Translational-stage models emphasize movement from discovery to clinical application, implementation, and population-health impact [3]. The present framework brings these literatures into a translational pharmacology problem: how information dynamics can distort the perceived maturity of therapeutic evidence, with consequences for clinical prioritization, therapeutic uptake, and public-health behavior.

## Translational comparators during COVID-19

Ivermectin was not the only pandemic therapeutic candidate whose interpretation depended on alignment among evidentiary stage, pharmacologic plausibility, clinical context, and public meaning. Hydroxychloroquine illustrates premature public confidence: early mechanistic plausibility and limited clinical enthusiasm moved rapidly into widespread attention before larger randomized trials failed to demonstrate meaningful benefit [5]. Corticosteroids illustrate stage- and phenotype-dependent interpretation. Early observational studies raised concerns that corticosteroid therapy might delay SARS-CoV-2 clearance [6], whereas later randomized evidence demonstrated mortality benefit from dexamethasone in selected hospitalized patients receiving oxygen or ventilatory support [7].

More upstream predictive and computational studies provide another orientation point. Single-cell transcriptomic and drug-prediction analyses, for example, identified CNS susceptibility pathways and included a drug-prediction result suggesting that (-)-catechin might reduce the effects of COVID-19 on the nervous system [8]. Such studies may contribute to hypothesis generation, but they remain early translational signals unless supported by exposure plausibility, experimental validation, and controlled clinical evidence. These comparators are not equivalent cases, and they need not be. Their purpose is to show that misalignment can arise when mechanistic plausibility is treated as clinical confidence, when observational associations are read without adequate attention to disease stage or phenotype, or when computational predictions are treated as therapeutic leads. Ivermectin remains the focal case because multiple forms of misalignment converged simultaneously.

## Ivermectin as a low-confidence translational signal

Drug-repurposing signals often emerge from legitimate preclinical observations. Caly et al. reported that ivermectin inhibited SARS-CoV-2 replication in vitro, with substantial reduction in viral RNA after exposure to 5 µM ivermectin and an inferred inhibitory concentration in the low micromolar range, approximately 2–3 µM [9]. That finding was meaningful as a screening observation and sufficient to justify further investigation. It did not establish clinical plausibility.

In translational pharmacology, a cell-based antiviral signal is not self-interpreting. Its clinical meaning depends on whether pharmacologically active concentrations can plausibly be achieved at the relevant site of action under clinically tolerable dosing. In the ivermectin case, the initial antiviral report was followed by pharmacokinetic and pharmacologic analyses that substantially constrained the direct antiviral hypothesis [10–12]. Early analyses estimated human plasma exposures in the tens of ng/mL range, corresponding to total concentrations well below the low micromolar range, depending on dose and modeling assumptions [10–12]. This comparison is conservative because ivermectin is highly protein-bound, reducing free pharmacologically active drug [11]. Even when total plasma concentrations are considered, the in vitro activity range exceeded typical systemic exposures under approved oral antiparasitic dosing by one to two orders of magnitude, with a larger discrepancy when free rather than total drug concentration is considered.

This was not simply a standard-dose limitation. Guzzo et al. had reported that ivermectin was generally well tolerated in healthy adults at doses up to approximately 10 times the approved 200 µg/kg dose [13]. Subsequent COVID-19 pharmacokinetic analyses concluded that even exposures at or near this upper characterized range remained below the concentrations associated with in vitro anti-SARS-CoV-2 activity [10, 11].

The relevant question was not whether ivermectin could be detected systemically, but whether unbound drug at relevant target sites could plausibly approach concentrations associated with antiviral activity. Schmith et al. argued early in the pandemic that approved ivermectin dosing was unlikely to achieve such exposure [10]. Peña-Silva et al. highlighted similar pharmacokinetic limitations [11]. Arshad et al. extended this logic across candidate drugs, emphasizing that repurposing should be prioritized on the basis of achievable plasma and target-site concentrations rather than in vitro activity alone [12].

The value of such analyses was not limited to retrospective interpretation. Under emergency conditions, pharmacologic plausibility can serve a triage function. Arshad et al. analyzed 56 drugs with reported in vitro anti-SARS-CoV-2 activity and found that only a minority were expected to achieve plasma exposures plausibly consistent with antiviral activity under approved human dosing [12]. Even a simple comparison between experimentally active concentrations and achievable human exposure can help determine whether a signal warrants accelerated clinical investigation, lower-priority exploratory study, or an explicit mechanistic rationale before further translational investment.

These analyses did not disprove the in vitro finding. They changed the evidentiary posture of the hypothesis. Before plausibility assessment, ivermectin could be described as an antiviral signal warranting further study. After plausibility assessment, the signal required a constrained interpretation: standard-dose direct antiviral efficacy appeared pharmacologically challenging, and further investigation required an explicit rationale for how dose, tissue exposure, alternative mechanisms, or other factors might overcome that limitation.

This point is central to the stewardship argument. Pharmacologic plausibility does not supersede rigorous clinical evidence. Rather, it affects prior plausibility, investigative priority, and the evidentiary threshold required to interpret early clinical findings. When a proposed mechanism is exposure-constrained, positive early clinical results require heightened attention to dose rationale, target-site exposure, bias, and reproducibility.

The stewardship problem appears in the timing and circulation of these findings. The original antiviral signal and the pharmacologic analyses required for its interpretation appeared in separate publications and circulated with different visibility. During an emergency, that separation mattered. The in vitro finding could be cited as rationale while the exposure constraints that should have shaped expectations remained peripheral. The positive signal propagated more efficiently than the limiting conditions needed to interpret it.

## Early clinical evidence and evidence-base fragility

The early clinical literature did not resolve ivermectin’s pharmacologic constraints; it introduced another layer of uncertainty. Supportive studies were often small, heterogeneous, and methodologically limited, with variation in disease stage, dosing, treatment timing, concomitant therapies, endpoints, and standards of care. Some were open-label or lacked clear allocation concealment and blinding; others relied on soft or context-dependent outcomes.

Mixed early evidence is common in emergency clinical literatures. The deeper problem was that weak and heterogeneous studies were sometimes treated as sufficient grounds for therapeutic confidence despite unresolved pharmacologic concerns. When an intervention’s proposed mechanism is exposure-constrained, positive early clinical findings require close scrutiny of dose rationale, endpoint selection, bias, reproducibility, and consistency with prior plausibility assessments.

Subsequent data-integrity concerns sharpened this issue. Lawrence et al. argued that the ivermectin literature demonstrated the risks of relying on summary-data meta-analyses when unstable and variably reliable studies are synthesized under conditions of intense public demand [14]. Garegnani and colleagues similarly described how misleading conclusions could emerge from weak primary studies and inadequately disciplined evidence synthesis [15]. The updated Cochrane review found no reliable evidence supporting ivermectin for treating or preventing COVID-19 outside well-designed randomized trials [16].

The Elgazzar study became the most visible academic example because it influenced some early syntheses before serious concerns rendered it unreliable. The broader issue extended beyond any single paper. Apparent benefits often attenuated or disappeared when analyses were restricted to trials at lower risk of bias. The repeated claim that “many studies showed benefit” therefore conflated study count with evidentiary weight.

Carvallo et al. illustrate a related problem at the boundary between fragile clinical evidence and public-facing amplification [17]. Their prophylaxis report described zero infections among healthcare workers receiving topical/buccal ivermectin plus iota-carrageenan, a finding that required rigorous scrutiny of design, exposure plausibility, endpoint ascertainment, allocation, confounding, adherence, and reproducibility before being treated as clinically actionable. Its translational significance lies less in its evidentiary strength than in its later use: an extreme reported effect could be cited, amplified, and converted into practical confidence without sustained engagement with the methodological, pharmacologic, and operational constraints surrounding the hypothesis.

## Translational misalignment in the ivermectin case

The ivermectin episode illustrates how perceived therapeutic readiness can move ahead of evidentiary position. The initial antiviral rationale remained weak at the T1 boundary because achievable human exposure did not plausibly match concentrations associated with in vitro antiviral activity [9–12]. The T2 evidence remained unstable because early clinical studies were heterogeneous, methodologically fragile, and later affected by data-integrity concerns [14–16]. Yet ivermectin was promoted in some clinical and public settings as though it had reached practice-ready or population-level status.

This was not only a misinformation problem. It was a translational-stage problem. A low-confidence biological signal, constrained by pharmacologic feasibility and supported by fragile clinical evidence, acquired the appearance of clinical readiness outside the ordinary sequence of evidence maturation. That appearance mattered because it increased susceptibility to amplification systems capable of converting unstable findings into clinical pressure, prescription-seeking, off-label use, self-medication, and resistance to later corrective evidence.

The CDC’s August 2021 Health Alert documented one downstream consequence. U.S. outpatient ivermectin prescriptions increased from a pre-pandemic baseline of approximately 3,600 prescriptions per week to more than 88,000 prescriptions in a single week, accompanied by increased poison-control calls and adverse events associated with misuse [18]. These indicators do not merely show public misunderstanding. They show that perceived evidentiary maturity had become operationalized as therapeutic behavior.

## Evidence synthesis, parallel validation, and credibility acquisition

Parallel validation refers less to a set of individuals than to a set of transmission functions through which evidentiary status can be reinterpreted. In the ivermectin case, four functions are analytically important: clinical translation, evidence consolidation, interpretive reframing, and behavioral uptake. Each can move a low-confidence signal closer to clinical or public use without resolving the pharmacologic or methodological constraints needed to interpret it.

Clinical translation was visible when preliminary or contested evidence was rendered as protocols, testimony, or practice-facing recommendations. FLCCC protocols, Pierre Kory’s December 2020 Senate testimony, and the Kory et al. review are cited here as artifacts of that role rather than as biographical focal points [19, 20]. Evidence consolidation was visible when review and meta-analytic formats presented heterogeneous findings as cumulative support. The Bryant and Lawrie systematic review, meta-analysis, and trial sequential analysis was explicitly framed as informing clinical guidelines [21]. The Kory et al. review and the Bryant and Lawrie meta-analysis later received Expressions of Concern, and Rothrock et al. subsequently argued that pooled mortality claims were sensitive to fragile study selection, variable trial quality, and contested or unreliable studies [22–24].

Fragile primary evidence could enter the same pathway. The Carvallo et al. prophylaxis report described an extreme protective effect among healthcare workers, but its translational significance for this argument lies less in its evidentiary strength than in its later mobility: a striking claim could move from a methodologically fragile and pharmacologically constrained report into credentialed amplification while the constraints remained peripheral [17, 20].

The point is not that evidence synthesis, clinical advocacy, or public communication is inherently inappropriate. Rapid synthesis and urgent communication are essential during emergencies. The problem is that these forms can confer an appearance of maturity that the primary evidence base does not yet support. When low-quality, heterogeneous, or unstable studies are pooled, protocolized, or publicly translated under conditions of intense demand, they may accelerate translational misalignment rather than resolve it.

Quantitative analyses of online discourse and health behavior help show why this mattered. Hua et al. found that ivermectin discourse included claims of scientific and clinical support [25]. Ostrovsky and Parikh extend this point behaviorally, finding increases in ivermectin prescribing and searches for “buy ivermectin” during or after clusters of publications later retracted or marked by expressions of concern [26]. Perlis et al. found that self-reported use of ivermectin or hydroxychloroquine was associated with COVID-19 misinformation endorsement, lower trust in physicians or scientists, trust in social media, and conspiratorial thinking [27]. Together, these findings support the mechanism of parallel validation: unstable evidentiary claims gained behavioral force not because lay publics read the studies directly, but because mediated systems converted fragile findings into perceived therapeutic legitimacy.

Parallel validation should therefore be understood as the mechanism through which constraint separation becomes durable translational misalignment. Clinical authority translated preliminary evidence into protocols, testimony, and treatment recommendations. Evidence-synthesis authority converted heterogeneous and unstable studies into the appearance of cumulative support. Interpretive authority reframed uncertainty as suppression and methodological criticism as bias. Advocacy and media networks extended these interpretations to broader publics, where repetition, identity alignment, and institutional distrust conferred practical authority faster than standard translational adjudication could operate.

This temporal asymmetry is central to translational vulnerability. Constraint separation increases susceptibility to amplification systems; parallel validation determines whether and how rapidly misaligned signals translate into clinical, social, or population-level effects. Later corrective evidence then enters an environment in which uncertainty has already been converted into expectation.

## Later randomized trials and clinical adjudication

Larger randomized trials did not support earlier claims of clinically meaningful benefit. In the TOGETHER trial, ivermectin did not reduce hospitalization or prolonged emergency observation among high-risk outpatients with early COVID-19 [28]. In ACTIV-6, a higher-dose regimen of ivermectin, 600 µg/kg daily for six days, did not meaningfully improve time to sustained recovery or other clinically important outcomes [29]. The higher-dose study was especially relevant because it addressed recurring claims that earlier negative trials reflected inadequate dosing.

From a translational perspective, these findings adjudicated a weakly supported hypothesis whose direct antiviral rationale had been exposure-constrained from the outset. Larger randomized trials tested whether preliminary signals persisted under more rigorous conditions. They did not.

Institutional caution emerged before all later trial evidence was complete. In March 2021, the World Health Organization advised that ivermectin should be used for COVID-19 only within clinical trials because evidence remained inconclusive [30]. Merck similarly stated that available data did not support meaningful efficacy against COVID-19 and that available safety data were insufficient [31]. These responses are better understood as standard translational caution than as suppression.

The persistence of ivermectin advocacy after these developments highlights a problem for downstream correction. Once a therapeutic claim acquires practical authority through parallel validation, contrary evidence may not function as a stopping signal. It may instead be reinterpreted as evidence that the wrong dose was tested, the wrong endpoint was chosen, the wrong institution was trusted, or the therapy was not given a fair opportunity. This adaptive quality of parallel validation increases the importance of upstream stewardship, before a low-confidence signal acquires credibility sufficient to sustain successive reinterpretations.

## Translational stewardship as publication-ecosystem responsibility

The ivermectin episode supports a specific lesson: rapid therapeutic repurposing should be accompanied by stronger mechanisms for communicating evidentiary stage, pharmacologic plausibility, and uncertainty before preliminary findings acquire practical authority. Translational stewardship names this responsibility. Its core principle is that critical translational constraints should be encoded, preserved, and propagated alongside therapeutic signals.

This principle does not require suppressing early signals, delaying publication, or discouraging exploratory research. It asks that known or readily answerable constraints be visible when therapeutic significance and investigative priority are assigned. For consequential translational signals, pharmacologic plausibility and translational feasibility deserve treatment analogous to interpretive requirements already accepted elsewhere in scientific publishing: clinical trials address limitations and uncertainty, observational studies address confounding, and meta-analyses address heterogeneity and risk of bias. Reports of therapeutically relevant repurposing signals, especially under conditions of public-health urgency, should similarly address exposure feasibility, target-site plausibility, mechanistic uncertainty, and expected translational barriers when those issues are central to interpretation [32, 33].

This responsibility should not fall solely on authors of early laboratory reports. The publication ecosystem shapes how findings acquire significance. Journals, editors, reviewers, commentators, and evidence-synthesis groups help determine whether plausibility constraints remain visible or become peripheral. Under emergency conditions, editors and reviewers might ask whether key translational questions are already answerable: Are active concentrations achievable in humans? Are there existing pharmacokinetic data? Are there known tissue-distribution constraints? Are there mechanistic reasons to doubt translation? The expectation would not be that authors resolve every question, but that relevant constraints be identified and linked to the signal.

In emergency repurposing, plausibility constraints also have a triage function. They help determine whether a signal should move rapidly toward clinical testing, remain a lower-priority exploratory lead, or require a specific dose, tissue-exposure, or mechanistic rationale before substantial resources are committed. This function is especially important when clinical trials are time-consuming, expensive, and dependent on finite patients, infrastructure, and public attention.

Publication incentives make this especially important. Positive preclinical signals are publishable, memorable, and easily summarized. Constraints often appear as caveats, limitations, or less exciting follow-up analyses. Yet a plausibility analysis suggesting weak likelihood of clinical translation may be highly informative. Publication bias toward positive findings can distort the available evidence base and affect later synthesis, especially when negative or constraining findings receive less attention [34]. When exposure feasibility, target-site plausibility, or dose rationale materially affects investigative priority, the resulting constraint should be treated as a translational finding in its own right rather than as a peripheral limitation.

Several practical responses follow. Reports of in vitro activity for repurposed drugs should, where feasible, compare experimentally active concentrations with achievable human exposure. Early-stage candidates should be described by translational stage. Early clinical studies with therapeutic implications should include structured limitations addressing randomization, blinding, allocation concealment, endpoint stability, preregistration, dose rationale, exposure plausibility, and risk of bias. Journals and evidence-synthesis groups should also develop faster mechanisms for linking influential claims to later methodological concerns, data-integrity issues, or plausibility assessments.

This makes translational stewardship closer to early encoding than later correction. It does not depend on predicting which signal will later become socially consequential. Instead, it treats contextualizing constraints as part of how translational information should be packaged when therapeutic significance is assigned. In this respect, stewardship is aligned with reporting-reform traditions that seek to standardize the information necessary for interpretation at the time of publication, rather than relying on later correction after claims have circulated.

More broadly, translational stewardship targets an upstream point of inflection: the moment when credibility is being assigned to a therapeutic signal. The aim is not to predict which claims will later become socially consequential, but to ensure that plausibility constraints are encoded before credibility becomes detached from evidentiary position.

Such stewardship would not make pharmacologic plausibility trump empirical clinical evidence. It would help determine the evidentiary threshold required to interpret that evidence. When a proposed mechanism is exposure-constrained, positive early studies require especially careful scrutiny before they can support therapeutic confidence. When a plausibility analysis suggests low likelihood of translation, later studies should explicitly address how their findings relate to that constraint.

Pandemic conditions demonstrated that translational medicine can no longer assume that evidentiary adjudication will precede widespread therapeutic operationalization. Scientific findings now move through a broader information ecosystem in which citations, preprints, media attention, advocacy networks, and alternative evidence syntheses can confer legitimacy independently of the traditional pace of evidentiary maturation. Translational stewardship attempts to preserve alignment between plausibility and credibility before that alignment is disrupted.

## Conclusion

The ivermectin–COVID-19 episode exposed a pandemic-amplified translational vulnerability that extends beyond the failure of a single therapeutic candidate. A legitimate but exposure-constrained preclinical signal, fragile early clinical evidence, and unstable synthesis practices contributed to apparent therapeutic consolidation before reliable confirmation existed. Later randomized trials and institutional caution did not fully restore evidentiary alignment because the claim had already acquired broader clinical, social, and interpretive authority.

The central lesson for translational medicine is that evidence generation and evidence communication cannot be cleanly separated during public-health emergencies, and perhaps not under contemporary information conditions more generally. A therapeutic candidate’s evidentiary stage is itself critical information. When that stage is poorly communicated—or displaced by parallel systems of validation—preliminary findings can acquire clinical and social authority disproportionate to their pharmacologic and evidentiary support.

Translational stewardship provides a constructive response. It does not seek to prevent weak signals from entering the literature. It seeks to ensure that assessments of translational plausibility accompany those signals before credibility becomes detached from feasibility. In this sense, stewardship is not simply a downstream corrective practice; it is an upstream effort to encode the constraints needed to preserve evidentiary meaning as claims move from discovery toward application. Future emergencies will likely reproduce similar pressures. Translational medicine should prepare to generate evidence rapidly while preserving the constraints needed to interpret that evidence with equal clarity.

## Data Availability

Not applicable.

## Declarations

### Use of AI-assisted technologies

During drafting and revision, the author used OpenAI ChatGPT (GPT-5.5 Thinking; accessed May, June 2026) for editorial assistance on the final draft to review for clarity, proofreading errors, and formatting support, and to assist with source discovery. All sources were independently verified, reviewed, and employed in the text. The author takes full responsibility for the manuscript.

### Authors’ information

The author is a retired Lecturer in Comparative Media Studies/Writing at the Massachusetts Institute of Technology, where he taught and researched scientific and technical communication and analysis in the Writing, Rhetoric, and Professional Communication program, working alongside technical faculty across disciplines, including bioengineering where he helped usher therapeutic projects from bench to bedside.
